# Investing in human capital in the era of European Universities: A comparative analysis of skill mismatches across the regions of the ERUA countries

**DOI:** 10.12688/openreseurope.16470.1

**Published:** 2023-10-02

**Authors:** Effie Emmanouil, Kostas Gourzis, Kostas Boukouvalas, Stelios Gialis

**Affiliations:** 1 Geography Department, University of the Aegean, Mytilene, Greece, 81132, Greece

**Keywords:** Human Capital Theory critique, Geographical Political Economy, comparative analysis, highly educated employees

## Abstract

Investment in human capital in the form of education and its returns for workers in terms of better-quality jobs remains a contested issue in the literature. While some postulate that such an investment leads to marginal productivity increments, others refute this hypothesis arguing that it overlooks crucial political economic factors. To contribute to ongoing debates, this paper provides a comparative analysis at the regional level, covering the period between 2009 and 2021 and 5 EU countries (Greece, Bulgaria, France, Germany, and Denmark), putting particular emphasis on the regions that are associated with the countries of the European Reform University Alliance (ERUA). Specifically, the analysis calculates highly educated individuals employed in low-, medium-, and high-skill occupations, and then identifies the relative regional concentrations of each (mis)match using the Location Quotient Index. Findings highlight the uneven geography of skill mismatches, as robust and innovative economies tend to allocate their labour force across occupations more efficiently than peripheral regions. Discussing our key findings through the lens of Geographical Political Economy, we point to five interconnected determinants of human capital allocation: national economies’ dynamism, regional industrial specialisations, degree of urbanisation, local institutional capacity, and demographics. Concluding, five sets of policy recommendations are proposed aligning with the EU New Skills Agenda and the European Universities Initiative.

## Plain language summary

The economic downturn of the last fifteen years, beginning with the 2008/09 Global Crisis up to the currently unfolding energy crisis, have shaped and accelerated the European Commission’s efforts towards the digital and energy transitions (often referred to as twin transitions), paying particular emphasis on workforce’s skills and education. The EU Skills Agenda sets ambitious targets to ensure access to education and lifelong learning in all regions of the Union. Adopting a comprehensive approach which incorporates institutional, evolutionary, and political economic influences, this paper advances the study on how investment in human capital (in the sense of education) is reflected in the level of skills used by workers in their occupations by examining their spatially uneven distribution.

To do so, it identifies the regional concentrations of highly educated workers in occupations requiring a high-, medium-, and low-level skillset. Data cover the countries (Greece, Bulgaria, France, Germany, and Denmark) and regions where the university departments comprising the European Reform University Alliance (ERUA) consortium are located (North and South Aegean, Île de France, Yugozapaden, Freiburg, and Sjælland respectively). Specifically, the consortium currently implements the ReERUA Horizon Project (Grant agreement ID: 101035808), in the frame of which the analysis at hand is conducted.

The study highlights five distinct yet interconnected parameters that directly influence human capital allocation: national economies’ dynamism, regional industrial specialisations, degree of urbanisation, local institutional capacity, and demographics. Being the result of a wide array of factors, we stress that skills are distributed quite unevenly across space. Drawing from the above and based on the EU's new skills Agenda, the study concludes proposing five actions for the ERUA regions (that can be applied to other contexts as well) towards a more effective allocation of skills (functionally and geographically-wise) and along a more equitable socioeconomic growth.

## Introduction

With the 1970s crisis of Fordism leading to long processes of industrial restructuring and deindustrialisation, workforce skills have come to the forefront ( Thrift, 2008). More specifically, amid an unstable and often recessive conjuncture which has been exacerbated by the successive crises of the last decade (the 2008/09 Global Crisis, the COVID-19 pandemic, the ongoing energy crisis), employees and regulatory bodies keep stressing the importance of new hard skills—often associated with the digital and green transitions—as well as soft skills related problem-solving and adaptability to changing conditions ( McGuinness *et al.*, 2018). Indicatively, the European Commission (EC) has increased its focus on measures that boost workforce skills, as reflected in the "Agenda for Skills" adopted in 2016 ( EC, 2016), the new "Agenda for Skills for Sustainable Competitiveness, Social Justice and Resilience" ( EC, 2020), and the proclamation of 2023 as the ‘European Year of Skills’. The EC has set ambitious targets for upskilling and reskilling the workforce with an implementation horizon of 2025, and a closer look reveals that its policy toolkits strongly resonate with the basic tenets of Human Capital Theory (HCT from now on; Becker, 1964; Mincer, 1958; Mincer, 1974; Schultz, 1961; Schultz, 1970). In this context, investment in education is not only presented as a way for people to improve their skills and competencies so as to position themselves better within the labour market, but also as a means for upgrading human capital overall and produce economic growth ( EC, 2016; EC, 2020).

In its fundamental narrative, HCT postulates that education increases the marginal productivity of labour (generating growth in collective terms) and eventually leads to higher income (for individuals). However, such simplistic and positivist accounts have come under question especially since the crisis of Fordism in the 1970s and the subsequent complex disruptions in Global Value Chains (GVCs from now on). Specifically, critique coming from class-focused views highlights how education reproduces unequal societal relations ( Galiakberova, 2019), while Geographical Political Economy accounts point that decisions of local, non-business actors in relation to education are not always rational, and are influenced decisively by globalisation trajectories, producing in this way spatial unevenness ( Teixeira, 2022). Overall, a good part of mainstream human capital-related theorisations is not grounded on empirical research (see Marginson, 2019; Nafukho *et al.*, 2004; Tan, 2014; Teixeira, 2022), while research on skill supply and demand has produced quite contradictory findings (see Brunello & Wruuck, 2021; McGuinness *et al.*, 2018; Sala, 2011).

In an effort to offer a fresh eye on the literature’s contradictory results and fill its gaps, the paper at hand follows highly educated workers and examines where they tend to be employed in high-, medium, and low-skill occupations. To do so, it builds upon a conceptual framework that combines Geographical Political Economy and human capital-related theorisations, and then conducts a comparative empirical analysis that utilises the Location Quotient Index (LQ from now on) for the period 2009–2021 and on 5 countries (Greece, Bulgaria, France, Germany, and Denmark). The selected study countries relate directly to the European Reform University Alliance (ERUA), which currently implements the ReERUA Horizon Project. Specifically, the Alliance comprises universities located at North and South Aegean (in Greece), Île de France (in France), Yugozapaden (in Bulgaria), Freiburg (in Germany), and Sjælland (in Denmark); throughout the analysis, particular emphasis is put upon these regions. Our study identifies five parameters influencing workforce skills’ geographical and functional allocation—economic dynamism at the national level, regional industrial compositions, level of urbanisation, demographics, and institutional characteristics—concluding that the mechanisms of human capital development tend to reproduce uneven development rather than ameliorating spatial inequalities. The above outline our paper’s multifarious contribution. On the one hand, it offers valuable insights into the negative externalities associated with skill mismatches in the regions of five countries representing the different socio-economic models within the EU (Nordic, Continental European, Mediterranean and post-socialist; Farkas, 2011), going beyond the basic theses of prevailing theories such as HCT; in this way, it takes a distinct position within the ongoing debates. At the same time, it strengthens ERUA through its study of the skill gaps between the consortium's associated regions that can eventually help bridge them, ultimately making in this way a crucial contribution to EU initiatives such as that of the European Universities.

The paper is structured as follows: the next section provides a theoretical framework on education and skills in the labour market and develops a conceptual framework based on approaches from Geographical Political Economy and elements from HCT, the Job Competition Model, and Assignment Models. Then it lays out the methodology and presents the results of the empirical analysis. Finally, the key findings are discussed within the context of the relevant literature, while the paper closes with brief conclusions that include a set of policy recommendations.

## Human Capital and its uneven spatial distribution: Theoretical considerations and a tentative conceptual framework

Albeit HCT is a theory which appeared in the literature early after WWII, it still has a firm grasp on the public discourse and policy documents. Essentially, HCT was established as a theorisation of wage income as a function of education and work experience ( Mincer, 1958). According to it, educational attainment is positively correlated with income because education increases skills, which in turn increase productivity, and higher productivity is rewarded with a higher income ( Becker, 1964; Strober, 1990). Implicitly, the idea of self-investment in one’s own capital in the form of education makes graduates responsible for individual success or failure, and the state is supposed to intervene when inequalities increase ( Marginson, 2019). That is because education is an investment that is crucial not only for workers themselves, but also for entire economies ( Tan, 2014). Indeed, when the average income of educated people increases, this in turn increases the national average income and eventually creates a multiplier effect ( Pritchett, 2001). At the same time, extensive horizontal mismatches constitute a distortion in the ideal geographical and functional allocation of human capital, reducing the productivity of firms and economic systems in general, and more importantly, profoundly diminishing an economy’s capacity to adapt to gradual restructuring processes or sudden shocks ( Brunello & Wruuck, 2021).

The skills, competencies, and knowledge required are determined by the tasks each worker must undertake for their position and the industry in which they work ( Giouli *et al.*, 2021). There are two broad categories of skills. For one, hard skills, which are the skills required to perform a certain job; these are mostly acquired through formal education, (re)training, and on-the-job experience, thus they are measurable through academic degrees and various types of certificates. Examples of such skills include language proficiency, expertise on certain software, technical skills, etc. ( McGuinness *et al.*, 2018). On the other hand, soft skills (also known as non-cognitive skills or interpersonal and personal skills) complement hard skills and are basic competencies needed in the workplace, even if they are not tied to a specific job. Communication, teamwork, and organisational skills are among the soft skills most often required by employers regardless of specific occupations ( McGuinness *et al.*, 2018). Employers consider soft skills as an indicator of job performance, as they determine a person’s ability to lead and/or work in teams ( Hewitt, 2006), and thus, largely determine career success ( Watts *et al.*, 2008). Overall, the development of both hard and soft skills is closely linked to employment and income opportunities, both waged and self-employment ( Vinayan *et al.*, 2020).

Despite the undeniable importance of skills, however, there are often mismatches between their supply and demand, as labour markets are being imperfect in their nature ( Teixeira, 2022). Specifically, there are two broad types of mismatches. Horizontal mismatches refer to cases where workers are engaged in activities outside their field of education and/or skills. For their part, vertical mismatches refer to cases where workers engage in activities that do not match their skill level. Although mismatches refer to both overqualification and underqualification, the paper at hand we will focus on the former. Overskilled workers tend to earn less than those who are in a position matching their skills, tend to have lower satisfaction levels, and are overall more inclined to quit their job. Importantly, they are less keen on acquiring new competences, albeit their high education does not protect them from skill obsolescence ( Somers *et al.*, 2019). Relatedly, overskilling worsens employment prospects in the long run, as it undermines incentives for young people to continue investing in human capital ( Cedefop, 2015). Many studies have shown that vulnerable groups such as migrants, women and young people are more often trapped in such situations ( Cedefop, 2014). For firms, overskilling indicates an untapped potential that is underutilised or not used at all, lowers productivity as their staff is dissatisfied, and limits their adaptation strategies as overskilled workers are reluctant to retrain ( Brunello & Wruuck, 2021). On a wider note, for national economies, firms’ lower productivity erodes competitiveness and investments in education are not returned into the economy ( McGuinness *et al.*, 2018).

In this context, HCT cannot provide a comprehensive analytical framework neither in terms of the geographical (dis)allocation of human capital ( Teixeira, 2022), nor in terms of individuals’ educational and career choices, which are not always based on rational processes ( Marginson, 2019). More specifically, HCT assumes that the human capital is allocated exclusively through labour market mechanisms, not taking political economic factors into account ( Teixeira, 2022). Namely, workforce skills sooner or later align with demand through labour force development systems, thus skill mismatches cannot exist in the long run ( McGuinness & Pouliakas, 2016). In this process, wider needs are translated into smaller scales through the operation of local labour regimes ( Jonas, 1996). As implied, HCT approaches the equilibrium between the supply and demand of skills within an “institutional vacuum” context, which is detached from processes outside the market and without broader power relations in place ( Psilos & Gereffi, 2011). In other words, HCT ignores the impact of space and the political economy, when in reality skills and labour are constantly influenced by broader processes including demographic and socio-economic changes, institutional settings, and the field of reproduction which includes family and peer pressure, cultural practices, etc. ( Peck & Smucker, 1998). The above attempt to outline how the development of skills is an inherently social process, brought about and maintained by hierarchical power relations within and outside labour markets ( Galiakberova, 2019). The influence of powerful non-entrepreneurial actors is key in shaping human capital, as fittingly described by theorisations around Local Labour Regimes ( Werner, 2019). Similarly, labour force development is not an equal opportunity field, but rather depends on characteristics such as class, race, gender, ethnicity and migrant status; in fact, labour force development plays a key role in perpetuating—not only social inequalities but also—labour market segmentation ( Teixeira, 2022). Lastly, crises and recessions have a decisive impact on the development of skills. Indicatively, the 2008/09 Global Crisis led to a concerted slashing of public spending on education, which profoundly altered human capital in the following years ( Gourzis & Gialis, 2019). All the above mark clear that the return of investments in education, whether at the scale of an individual or of a whole state, can be quite unpredictable.

Moreover, the above imply that many reproducing mechanisms related to human capital fuel spatial inequalities by favouring economic stability and growth for certain regions at the expense of others ( Teixeira, 2022). Specifically, with capital- and technology-intensive activities tending to concentrate in already advanced regions, new rounds of uneven development unfold ( Vinuela-Jimenez *et al.*, 2010). In this process, large metropolitan areas with a diverse economic base concentrate human capital, cutting-edge technology, and finance-related activities, while economically backward and declining regions become even more disconnected from knowledge and innovation ( Feldman *et al.*, 2021). Against this narrative of uneven development, many theories have argued that developed regions may tend to attract activities, workforce, and capital as they further industrialise, but they also do spill over positive effects to their surrounding areas ( Boudeville, 1968; Perroux, 1955). In fact, theoretical models such as the “poles of growth” have been well-established and dominant in policy and scientific debates since the first post-war decades ( Christofakis & Papadaskalopoulos, 2011). Countless strategic plans have been devised based on such theorisations, aiming at transforming agglomeration tendencies into a competitive advantage, with examples coming from many different geographical contexts including Germany, France, and Greece ( Hadjimichalis, 1992; Leontidou, 1990). After the end of Fordism, however, Growth Poles Models started losing their grip as they proved to be largely unsuccessful: conditions did not improve for most lagging regions, while large urban areas also suffered a number of negative externalities due to unchecked population growth and piling of activities ( Christofakis & Papadaskalopoulos, 2011). Nevertheless, new approaches in economic geography continue to emphasise the formation of dynamic urban centres due to their key role in the global economy and their spillover qualities ( Florida *et al.*, 2008; Fujita & Mori, 1996; Fujita *et al.*, 1999); as becomes clear, this fuels new rounds of Fordist-influenced spatial organisation ( Krugman & Venables, 1996).

In our view, such approaches and the policy responses they inspire reinforce the polarisation of economic growth right from the outset. As a result, it is not surprising that educational and employment opportunities still vary greatly across regions ( Fratesi & Rodríguez-Pose, 2016; Iammarino *et al.*, 2019). Certain regions that are locked into a positive path dependency, namely a process which reinforces virtuous cycles of regional growth path, while others are locked in their rigidities including outdated technology and institutional weaknesses, which reinforce vicious cycles of regional vulnerability. Moreover, the latter often find themselves deeper into negative path dependencies after recessive periods, as they have no capacity to absorb shocks and adapt to a new reality ( Rodríguez-Pose *et al.*, 2023). Having said the above, there are cases of regions reinventing themselves and carving new development pathways, revitalised and repositioned in emerging global production networks and chains of value added ( Boschma, 2015). Still, the norm for most already vulnerable regions is that they prove unable to create knowledge-intensive and high-tech jobs for high-skilled workers. These regions are typically peripheral, rural and less developed ( MacKinnon *et al.*, 2022). Lastly, it must be stressed that even within regions that have developed self-sustaining processes of growth stark inequalities emerge, as different fractions of capital seek for both cheap and skilled workers ( Teixeira, 2022). Importantly, such labour market segmentation is sustained by dual human capital development mechanisms; namely, next to a robust education system, a training system that focuses more on vocational training and apprenticeship initiatives is formed, serving as a channel for precarious labour ( Teixeira, 2019). The deepening inequalities within affluent regions is an emerging topic in the literature, with many attributing the rise of populism to this phenomenon ( Rodríguez-Pose *et al.*, 2023).

## Methodological remarks

The aim of this paper is to study the spatialities of the matching of highly educated workers in different skill-level occupations. Specifically, we study the regional (NUTS-2 level) concentrations of workers with high levels of education in low, medium, and high skill occupations. The paper focuses on high education levels to examine the contested issue of overskilling, which refers to instances where a worker is not using their whole skillset in their job ( Brunello & Wruuck, 2021). The severe short- and long-term consequences of overskilling, as indicated in the previous section, underline the high scientific importance and policy relevance of our study’s objective.

**Figure 1.A.**
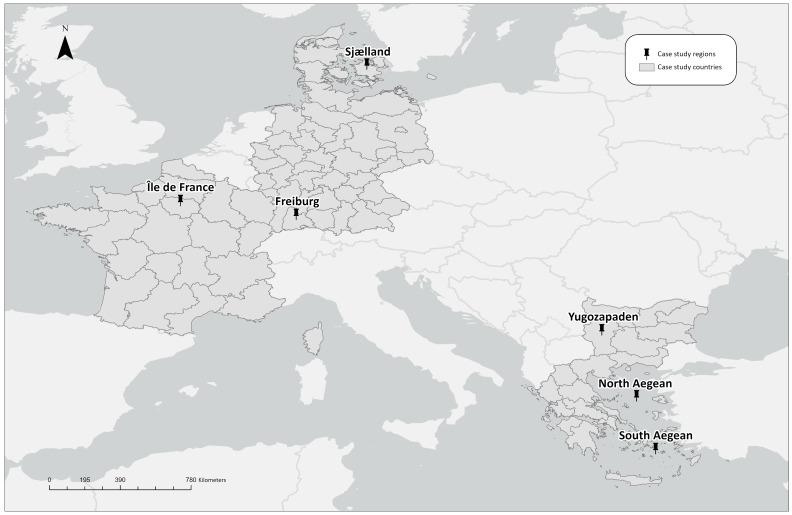
Regions related to the ReERUA Horizon Project. *Source:* Own elaboration.

The above research objective is scrutinised for five countries (Greece, Bulgaria, France, Germany, and Denmark), which were chosen on the basis that they stand for the four different socio-economic models that have been identified to currently co-exist within Europe: the Nordic, Continental European, Mediterranean, and post-socialist ones ( Farkas, 2011). Moreover, while both France and Germany fall under the Continental model, they are both analysed due to their stark differences in terms of their growth models, and particularly regarding their wage structures (which relate to this paper’s objectives; Hein & Vogel, 2008). This way, the analysis provides a view that can be representative of the EU as a whole. Moreover, throughout the analysis we focus on six regions which are specifically related to the ReERUA Horizon Project, in the frame of which the analysis at hand is conducted; these are the North and South Aegean regions in Greece, Île de France in France, Yugozapaden in Bulgaria, Freiburg in Germany, and Sjælland in Denmark (see Figure 1.A). These regions place particular emphasis on the knowledge that comes from their educational institutions; at the same time, they are developing strong knowledge networks within the ReERUA consortium, despite having very different socio-economic structures. As such, they constitute interesting case studies, especially when seen within their wider context.

The data were taken from the Eurostat Labour Force Survey upon request in July 2022.

The aforementioned analysis is conducted for two years (2009 and 2021), which essentially allow for a comparison between the onset of a period characterised by successive challenges for regional and national economies across the EU and the most recent available data. Specifically, this choice of years covers the Great Recession that followed the 2008/09 Global Crisis (lasting from 2009 until 2013–14 for most European economies), a period of partial recovery, and the two pandemic years (2020 and 2021; Herod *et al.*, 2021). As such, the temporal scope of this study constitutes an interesting case and reveals much about how regions received and adapted to recessive pressures.

To distinguish occupations by skill level, we use the International Standard Classification of Occupations (ISCO-08) of the International Labour Organisation (ILO) ^1^. Specifically, occupations are classified as high-skill if they correspond to ISCO occupational groups 1 to 3 (managerial and scientific positions), medium-skill when corresponding to ISCO levels 4 and 8 (intermediate level manual and non-manual positions), and low-skill if they correspond to ISCO level 9 (entry level manual and non-manual positions) (see also Table 1). To distinguish education levels, we use UNESCO’s (2012) classification. Specifically, education levels are distinguished into high if they correspond to ISCED levels 6 to 8 (tertiary education: from bachelor's degree to doctorate or equivalent), middle if they correspond to ISCED levels 3 to 5 (upper secondary to post-secondary education, including short tertiary education), and low if they correspond to ISCED levels 0 to 2 (pre-school to lower secondary). Data on the matching between highly educated workers and different skill level occupations at the NUTS-2 scale were retrieved from Eurostat. It must be mentioned here that data on highly educated workers in low-skill occupations displayed many missing values (as indicated in Figure 1.1a & Figure 1.1b, thus, results extracted in that part of the analysis must be approached with caution.

**Table 1.** Rates of workers in low, medium and high skills, per region, 2009 and 2021.

Region	Year	% Low skills	% Medium Skills	% High skills
BG34	2021	13,86	58,55	26,53
	2009	14,51	62	22,2
BG41	2021	7,85	44,55	47,6
	2009	11,06	49,67	38,4
BG33	2021	17,44	54,01	28,55
	2009	14,07	57,61	28,32
BG31	2021	12,96	60,27	26,77
	2009	13,05	61,52	25,43
BG42	2021	11,64	62,8	24,33
	2009	12,52	63,11	23,67
BG32	2021	10,73	61,98	27,29
	2009	11,44	60,77	27,79
DK04	2021	10,74	41,96	47,3
	2009	10,19	46,37	43,44
DK02	2021	10,83	45,25	43,93
	2009	8,94	46,98	44,08
DK01	2021	7,38	31,52	61,09
	2009	7,97	36,25	55,78
DK05	2021	11,91	45,86	42,22
	2009	10,7	48,64	40,66
DK03	2021	12,95	44,03	43,02
	2009	11,49	50,06	38,46
DE92	2021	8,46	45,32	46,22
	2009	7,93	46,29	45,78
DEB1	2021	9,6	48,78	41,62
	2009	9,32	50,74	39,17
DEC0	2021	7,79	48,52	43,69
	2009	9,21	51,02	39,77
DE26	2021	6,95	46,87	46,18
	2009	9,11	50,83	40,06
DE60	2021	5,12	37,23	57,65
	2009	6,58	40,22	53,2
DEB2	2021	12,89	39,27	47,84
	2009	9,06	50,08	40,86
DE50	2021	10,04	44,81	45,15
	2009	9,56	48,65	41,79
DED5	2021	4,81	41,12	54,07
	2009	8,16	48,21	43,63
DEB3	2021	7,44	45,36	47,2
	2009	8,92	46,77	44,31
DE30	2021	4,19	35,33	60,48
	2009	7,06	38,59	54,36
DE11	2021	7,85	44,67	47,48
	2009	7,91	46,83	45,26
DE80	2021	6,18	52,19	41,62
	2009	7,54	55,6	35,92
DEE0	2021	7,12	53,92	38,96
	2009	8,25	53,19	37,85
DEG0	2021	7,56	51,55	40,89
	2009	7,42	53,63	38,44
DEF0	2021	7,28	47,09	45,64
	2009	8,36	47,95	42,9
DE12	2021	7,78	43,89	48,33
	2009	8,33	46,5	45,17
DE21	2021	5,45	40,93	53,62
	2009	6,63	43,44	49,64
DE93	2021	8,44	48,72	42,84
	2009	8,68	50,65	39,94
DE94	2021	8,57	51,58	39,85
	2009	9,62	53,06	36,58
DE22	2021	7,04	52,22	40,74
	2009	9,88	55,55	34,57
DED4	2021	5,34	55,53	39,13
	2009	6,21	54,26	39,52
DE23	2021	7,87	51,29	40,84
	2009	9,28	54,68	36,03
DE25	2021	6,74	46,42	46,85
	2009	8,31	47,79	43,9
DE40	2021	6,02	49,86	44,11
	2009	7,18	51,48	41,34
DEA3	2021	7,58	47,63	44,79
	2009	8,36	51,13	40,5
DEA5	2021	8,08	48,99	42,94
	2009	9,24	49,83	40,93
DE73	2021	8,05	49,6	42,35
	2009	10,68	48,65	40,67
DE91	2021	7,32	47,04	45,64
	2009	8,54	50,49	40,97
DEA1	2021	6,78	45,73	47,49
	2009	7,49	47,87	44,64
DE24	2021	7,1	46,76	46,14
	2009	9,97	50,62	39,41
DE14	2021	7,81	46,68	45,52
	2009	9,09	49,38	41,53
DED2	2021	3,69	49,91	46,39
	2009	8,35	49,75	41,9
DE71	2021	7,68	42,98	49,34
	2009	7,57	43,75	48,68
DE13	2021	7,79	48,34	43,88
	2009	8,08	50,08	41,84
DE27	2021	7,31	50,58	42,11
	2009	7,9	54,06	38,04
DEA4	2021	8,61	48,92	42,48
	2009	10,14	50,61	39,25
DE72	2021	7,71	44,22	48,07
	2009	8,96	48,88	42,16
DEA2	2021	6,84	43,23	49,94
	2009	7,78	44,44	47,44
EL63	2021	6,23	71,36	22,4
	2009	4,95	65,64	28,78
EL41	2021	7,13	62,31	26,54
	2009	4,93	51,11	37,53
EL51	2021	7,81	65,77	19,59
	2009	4,96	63,82	27
EL62	2021	14,04	68,91	17,06
	2009	7,3	66,53	26,17
EL54	2021	10,9	63,25	25,85
	2009	6,19	65,78	28,03
EL30	2021	5,1	52,13	40,96
	2009	8,34	50,8	39,52
EL52	2021	6,3	61,34	31,01
	2009	6,49	58,1	34,66
EL53	2021	5,55	68,02	26,43
	2009	4,75	66,72	28,53
EL65	2021	6,55	70,5	22,95
	2009	6,87	68,08	25,05
EL43	2021	7,96	66,01	25,35
	2009	7,53	62,62	29,28
EL64	2021	10,22	69,98	19,8
	2009	9,23	64,08	26,68
EL61	2021	6,21	62,34	29,99
	2009	6,15	60,33	32,83
EL42	2021	5,86	65,97	27,01
	2009	9,67	61,19	29,14
FRI1	2021	8,5	44,63	44,98
	2009	8,08	48,99	42,35
FRF1	2021	10,11	46,34	43,55
	2009	7,32	50,89	40,92
FRB0	2021	10,35	46,47	42,52
	2009	8,3	50,41	40,19
FRC1	2021	10,25	44,24	45,51
	2009	9,75	54,71	35,55
FRG0	2021	8,95	48,41	42,13
	2009	8,03	55,26	36,26
FRJ2	2021	8,58	40,28	50,44
	2009	8,47	48,91	42,62
FRF3	2021	11,37	47,69	40,2
	2009	9,83	54,17	33,51
FRF2	2021	9,23	51,32	38,05
	2009	10,06	57,59	32,35
FRE2	2021	8,76	47,05	44,19
	2009	9,83	56,54	33,62
FRJ1	2021	9,14	41,16	48,9
	2009	9,79	50,11	38,1
FRD2	2021	8,24	47,83	43,92
	2009	11	52,66	36,34
FRE1	2021	10,11	43,53	46,37
	2009	10,39	50,27	39,34
FRK2	2021	7,86	41,04	51,1
	2009	10,47	49,26	39,94
FRK1	2021	8,87	52,12	39,01
	2009	9,98	51,14	38,88
FRC2	2021	6,89	54,85	38,26
	2009	8,87	55,5	35,63
FRD1	2021	7,86	50,79	41,35
	2009	8,79	54,72	36,49
FRL0	2021	9,4	40,67	48,39
	2009	12,41	48,48	37,93
FRH0	2021	9,58	45,62	43,19
	2009	8,79	53,07	37,14
FR10	2021	6,31	31,07	62,38
	2009	9,6	35,02	54,68
FRM0	2021	6,37	51,11	42,52
	2009	13,79	53,25	32,96
FRI2	2021	10,6	45,84	43,56
	2009	8,72	55,3	35,97
FRI3	2021	11,84	47,83	40,33
	2009	9,27	56,71	32,93

To identify regional concentrations of education/skills per level we calculate the Location Quotient Index (LQ). In general, the LQ highlights the relative concentration of a group within a region compared to its wider context; e.g., the share of industrial workers within a region divided by the respective share at the national level. Here, the LQ index is calculated by dividing the regional shares by the aggregate transnational share of the five countries under study. The LQ in the frame of this study shows the relative-to-transnational concentration of highly-educated employed persons in occupations requiring low [LQ(i)], medium [LQ(ii)] and high [LQ(iii)] skill levels, calculated through the following formulas ( 1.1, 1.2 and 1.3 respectively). This way, we find where each type of skill (mis)match is mostly concentrated. In our analysis, values between 0.85 and 1.15 are treated as being close to the average share, values above 1.15 indicate an overconcentration, while values below 0.85 show underconcentration.


$$LQ(i)=\frac{\frac{highly-educated\;employed\;persons\;in\;low\;skill\;level\;occupations\;in\;region}{employed\;population\;in\;region}}{\frac{highly-educated\;employed\;persons\;in\;low\;skill\;level\;occupations\;in\;all\;countries\;under\;study}{employed\;population\;in\;all\;countries\;under\;study}}\;(1.1)$$

$$LQ(ii)=\frac{\frac{highly-educated\;employed\;persons\;in\;medium\;skill\;level\;occupations\;in\;region}{employed\;population\;in\;region}}{\frac{highly-educated\;employed\;persons\;in\;medium\;skill\;level\;occupations\;in\;all\;countries\;under\;study}{employed\;population\;in\;all\;countries\;under\;study}}\;(1.2)$$

$$LQ(iii)=\frac{\frac{highly-educated\;employed\;persons\;in\;high\;skill\;level\;occupations\;in\;region}{employed\;population\;in\;region}}{\frac{highly-educated\;employed\;persons\;in\;high\;skill\;level\;occupations\;in\;all\;countries\;under\;study}{employed\;population\;in\;all\;countries\;under\;study}}\;(1.3)$$

Lastly, the specific characteristics attributed to regions throughout the analysis and discussion sections are based on insight provided by the Regional Innovation Scoreboard ( European Commission, 2022)

## An analysis of highly educated workers per occupational skill level from a cross-regional perspective

### Highly educated workers in low skill level occupations

LQ(i) shows the relative regional concentration of highly educated employed persons in low-skill occupations. In 2009, overconcentration of this phenomenon was mostly observed in Île de France (LQ=2.62) and in the southern regions of France, namely the agricultural regions of Midi-Pyrenees (LQ=3.42) and Auvergne (LQ=3.83), in the affluent region of Rhône-Alpes (LQ=2.36), and in the touristic region of Provence-Alpes-Côte d’Azur (LQ=2.52). Similar trends can also be observed in Thuringia (LQ=2.21), a region in Germany known for its university, the Greek capital region of Attiki (LQ=1.97), and the capital region of Bulgaria Yugozapaden (LQ=1.81). For 2021, we can see that in all French regions for which data are available LQ values are above 2.15. Other regions with particularly high LQ values are Hovedstaden (LQ=3.30) and Midtjyllan (LQ=3.25) in Denmark. Underconcentrations or close to the transnational aggregate one were not observed in the regions with available data (see Figure 1.1a and Figure 1.1b). 

**Figure 1.1a.**
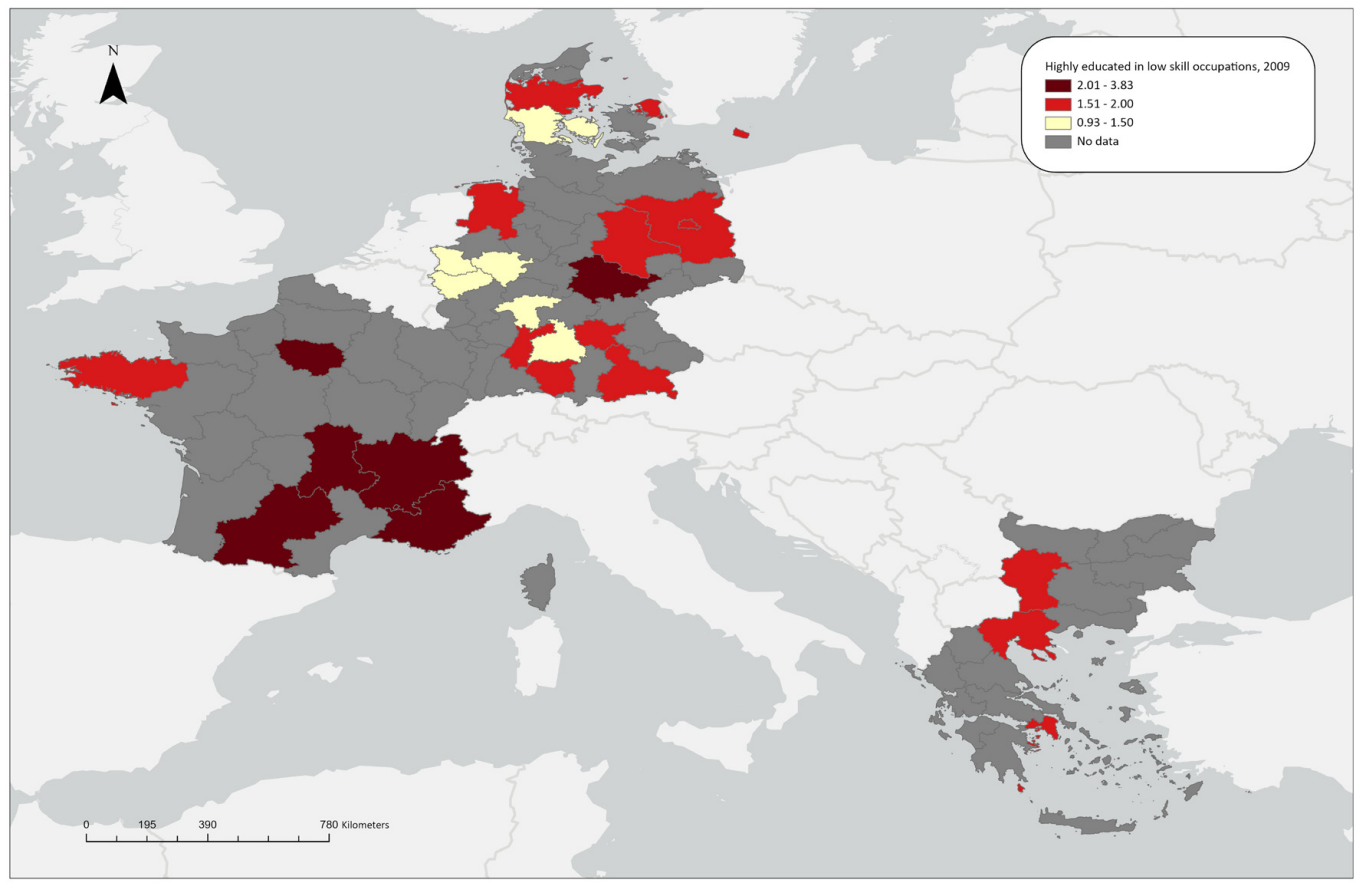
Over/under-concentrations of highly educated workers in low skill-level occupations, relative regional concentrations 2009. *Source:* Own elaboration.

**Figure 1.1b.**
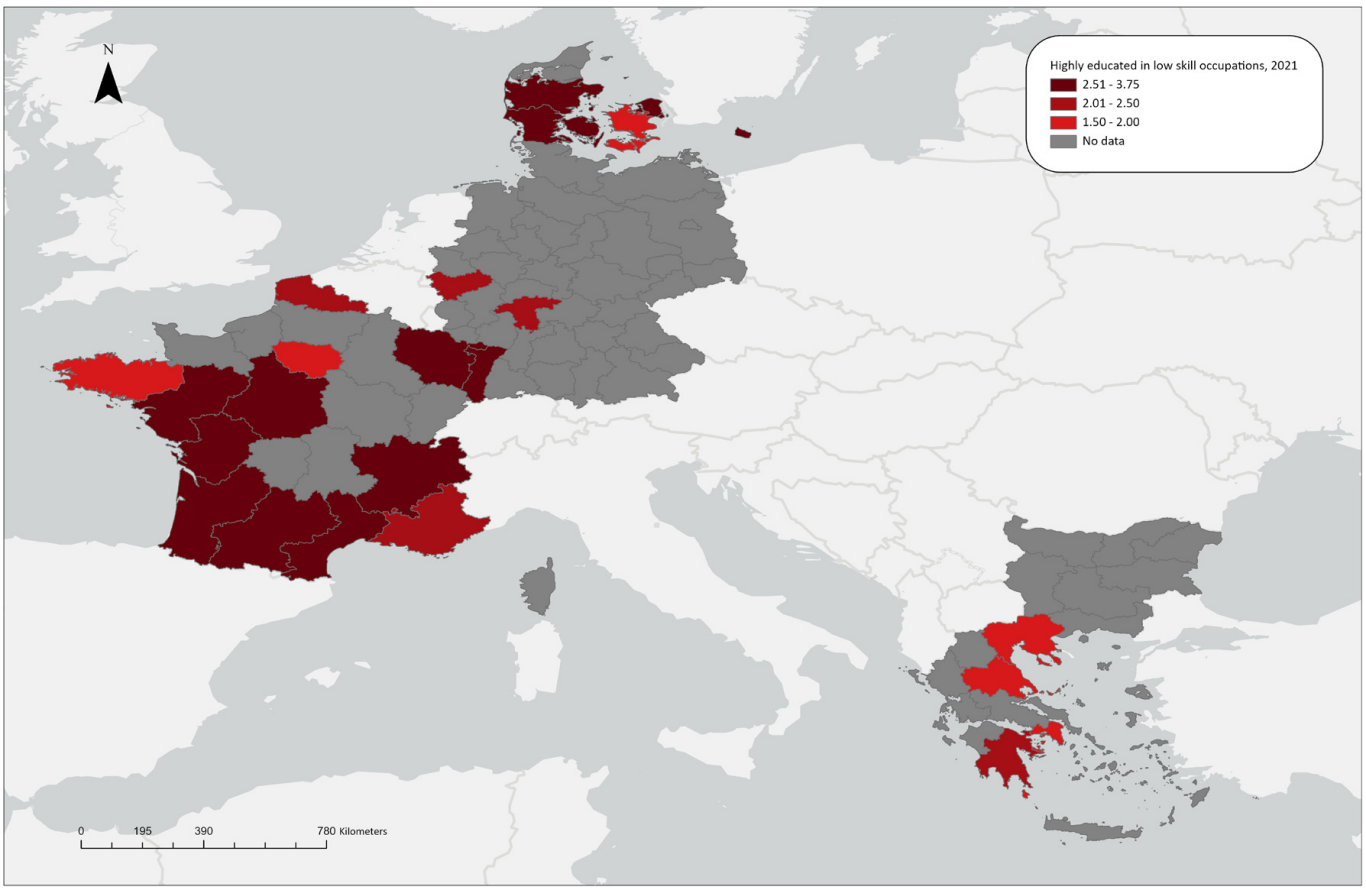
Over/under concentration of highly educated employed persons in low skill occupations, relative regional concentrations, 2021. *Source:* Own elaboration.

**Figure 1.2a.**
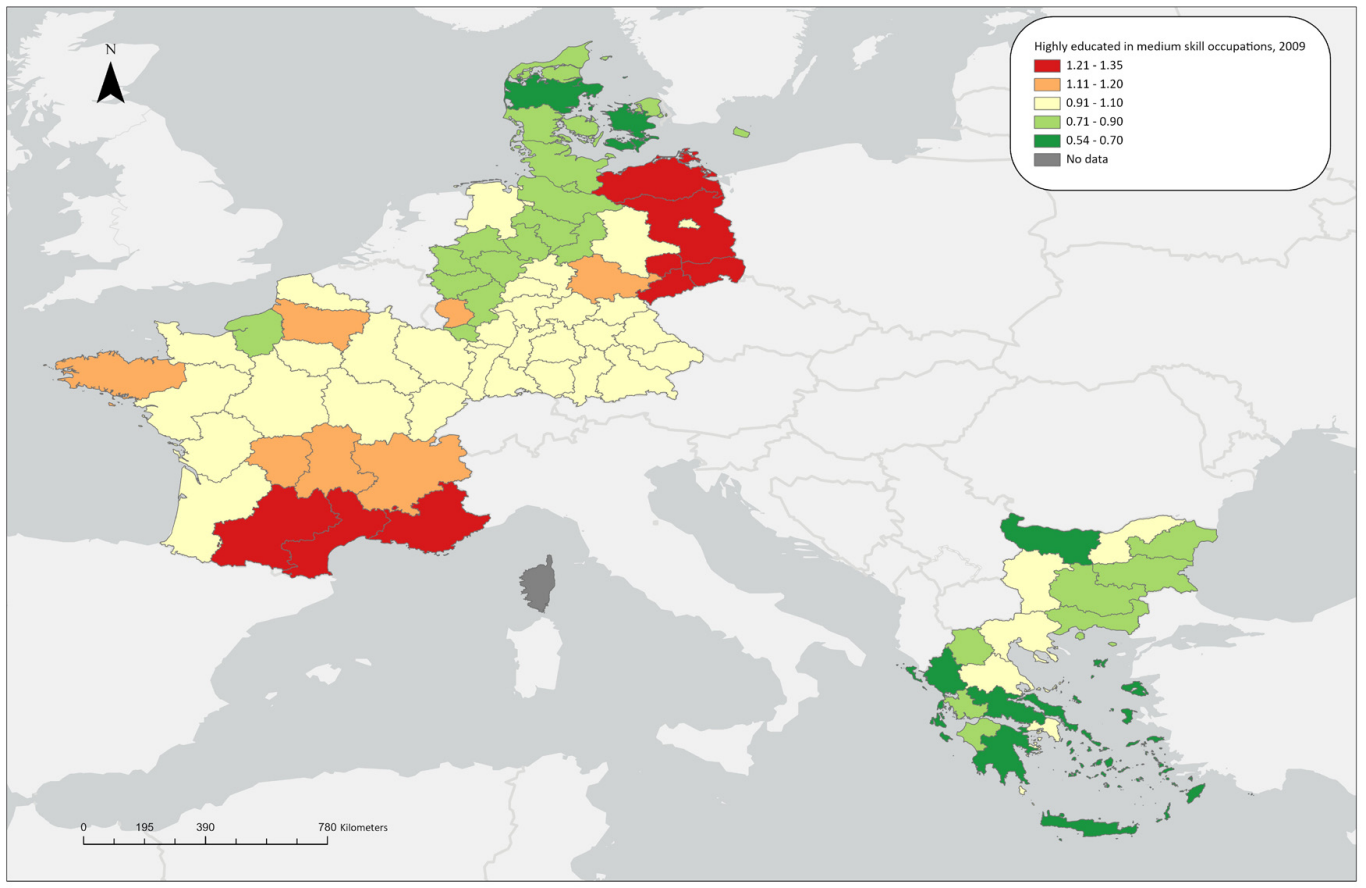
Over/under concentration of highly educated persons in middle skill occupations, relative regional concentrations, 2009. *Source:* Own elaboration.

**Figure 1.2b.**
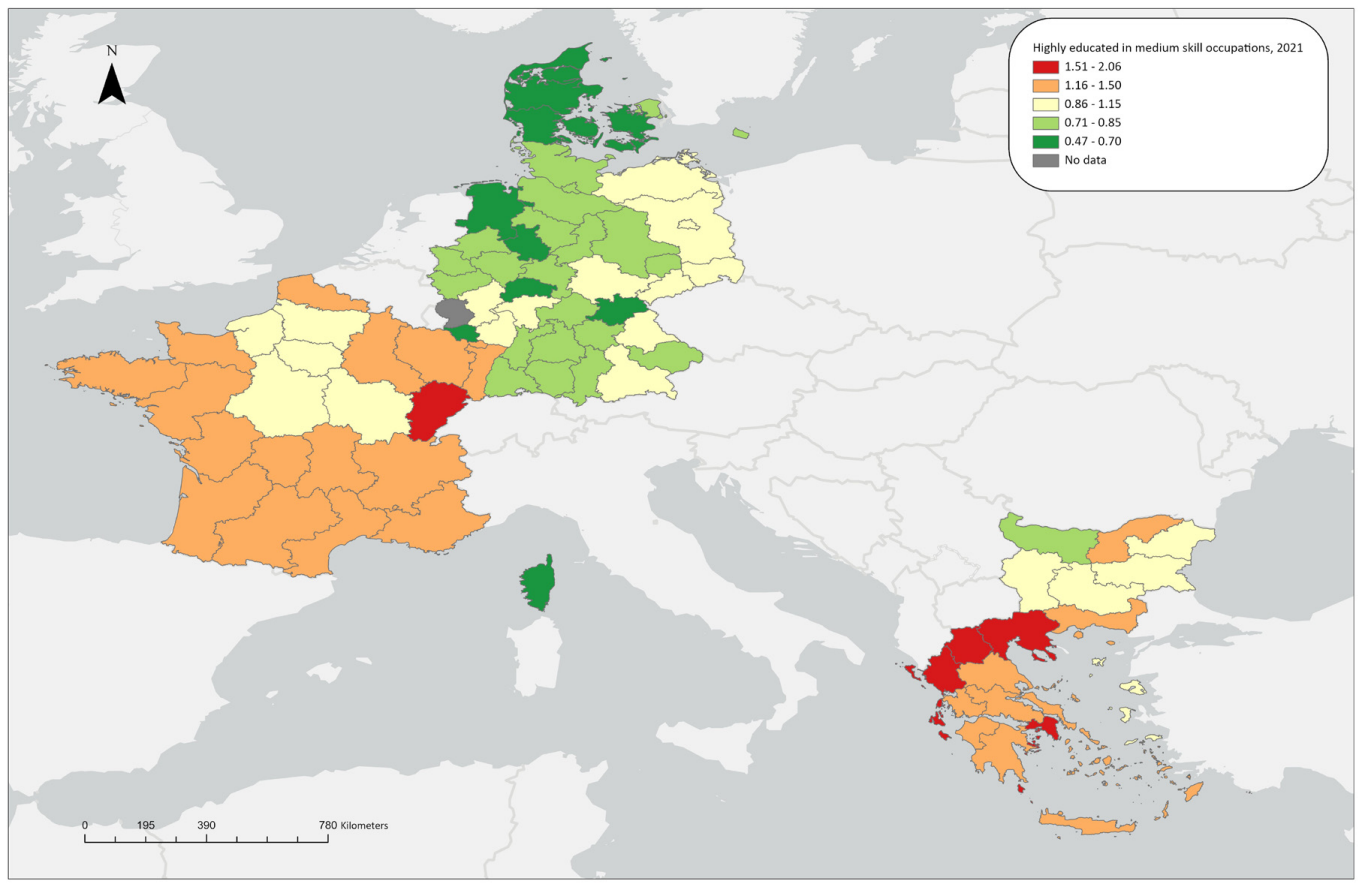
Over/under concentration of highly educated persons in middle skill occupations, relative regional concentrations, 2021. *Source:* Own elaboration.

**Figure 1.3a.**
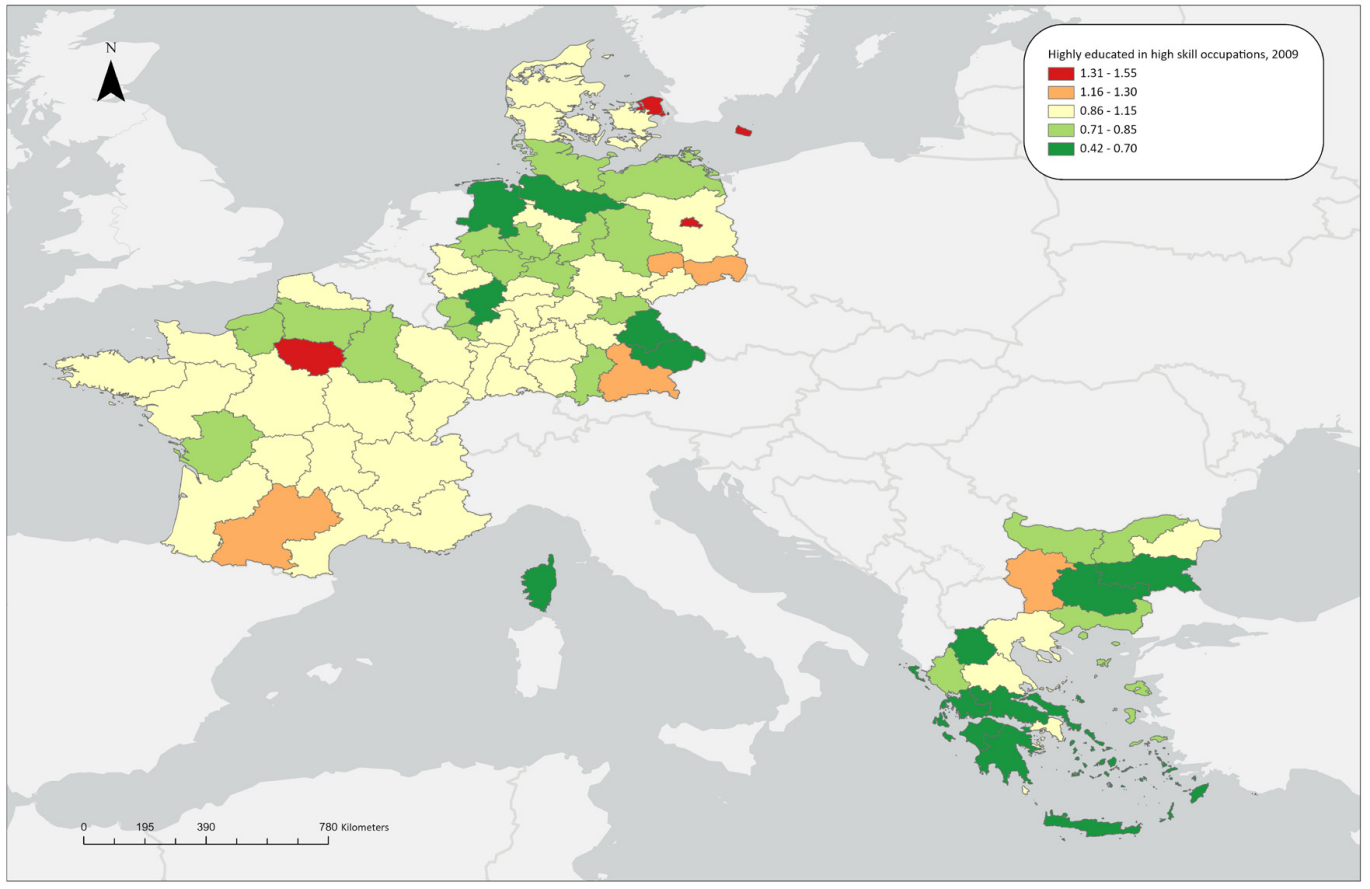
Over/under concentration of highly educated employed persons in high skill occupations, relative regional concentrations, 2009. *Source:* Own elaboration.

**Figure 1.3b.**
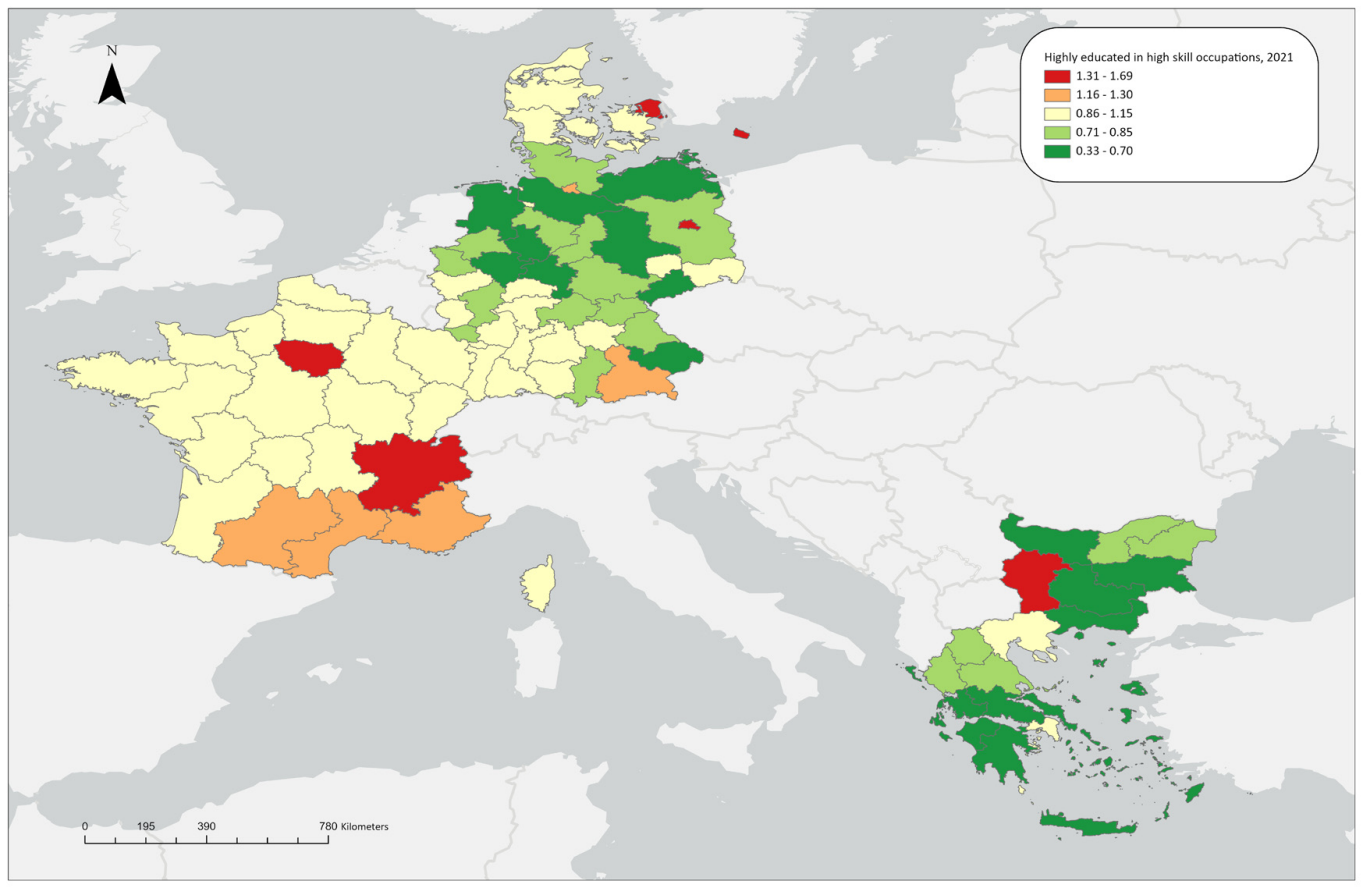
Over/under concentration of highly educated employed persons in high skill occupations, relative regional concentrations, 2021. *Source:* Own elaboration.

### Highly educated workers in medium skill level occupations

LQ(ii) for 2009 shows that five German regions (Leipzig, Chemnitz, Brandenburg, Dresden, Mecklenburg-Vorpommern and Dresden) stand out in terms of concentrating highly-educated workers in medium skill-level occupations, with LQ values around 1.30. Additionally, three regions in the southern part of France (Midi-Pyrénées, Languedoc-Roussillon, Provence-Alpes-Côte d’Azur) exhibit a similarly mild overconcentration. All other regions stay close to the aggregate average of the countries under study. In the regions related to the ReERUA Project, this type of matching is not extensive. Specifically, underconcentration is observed in North and South Aegean in Greece and Sjaelland in Denmark, while Freiburg and Île de France have values close to 1 ( Figure 1.2a).

In 2021, the overall number of regions exhibiting overconcentration increased. However, Attica stands out (LQ=2.06), as well as the other Greek regions show similar trends with the exception of the rural North Aegean (LQ=1.06). The German regions have low or close to the aggregate average shares, including Freiburg. The same applies for Denmark; indicatively, the highest value in the country can be found in Hovedstaden (LQ=0.82). In France, most regions exhibit an over-concentration; only seven regions are exceptions to this, having shares close to the aggregate average, including Île de France ( Figure 1.2b).

### Highly educated workers in high skill level occupations

For 2009, almost all capital regions of the countries under study exhibit notable overconcentrations (1.25 in Yugozapaden, 1.55 in Île de France, 1.43 in Berlin and 1.50 in Hovedstaden) except for Attica in Greece, whose share is close to the aggregate average. Most other regions in the countries under study display medium or low LQ values, with the exception of Leipzig and Midi-Pyrénées ( Figure 1.3a). This pattern remains almost unchanged in 2021, with the exception that Rhône-Alpes, one of the ‘four Motors for Europe’, also shows an overconcentration of highly educated workers in high-skill level occupations, illustrating the long-standing concentration of these workers in metropolitan, highly industrialised and research-oriented regions. Finally, as far as the ReERUA-related regions are concerned, the remote North and South Aegean exhibit a notable underconcentration (0.69 and 0.58 respectively), Sjælland and Freiburg exhibit average LQ values, while Île de France has the highest value (LQ=1.69) among all regions under study ( Figure 1.3b).

## Discussing the determinants of human capital allocation

Our analysis above has highlighted the uneven geography of skill and education levels’ matching across the regions under study. Here, drawing upon our findings, we will discuss the deeper causes of these socio-spatial inequalities, highlighting five distinct, yet interrelated parameters that directly shape the distribution of human capital: (a) the economic dynamism of national economies, (b) the particularities of regional industrial structures, (c) urbanisation levels, (d) local institutional structures and practices, and (e) demographics.

First, there are multiple indications that **dynamism of national economies** is a prime determining factor for the spatially uneven distribution of human capital and skills’ matching. In our study, robust national economies, both in terms of total and per capita GDP (Germany, Denmark, France), were found to overall exhibit more notable concentrations of highly educated workers in high skill positions than less dynamic ones (Bulgaria, Greece); Denmark had the highest GDP per capita in 2021 among the countries under study, being above the EU average by 33%, while Germany and France followed (with 20% and 5% respectively). Additionally, a closer look reveals that the former countries have oriented their production towards dynamic sectors comprising high added value manufacturing, financial services, and other specialised B2B services ( Herod *et al.*, 2021). Beyond exhibiting more efficient skill matching, countries with dynamic sectoral specialisations concentrate much higher shares of a high-skilled workforce in the first place ( Lee & Rodriguez-Pose, 2013). The reason that some countries can develop a workforce with a higher skill set whereas others struggle to follow, is their positioning within global value chains (GVCs). In one way or another, being embedded to some extent is common for almost all national economies ( Fernandez-Stark *et al.*, 2011), albeit whether this embeddedness benefits them ( Humphrey, 2004) or exposes them to external pressures ( Psycharis *et al.*, 2014) remains an open question. In general, it is commonly accepted that already advanced economies achieve a better positioning which brings upon them a series of positive outcomes, including being able to attract high-skilled labour, as they have the capacity to utilise it properly ( Fernandez-Stark *et al.*, 2011).

Beyond the national scale, positioning within global production networks (GPNs) translates in profoundly different ways at the regional level ( Fernandez-Stark *et al.*, 2011). Characteristically, stark inequalities among regions regarding skill and education matching were found in Germany and France despite being strong national economies, while instances of a great matching were identified within lagging national economies, as in the case of Bulgaria’s and Greece’s capital regions (Yugozapaden & Attica respectively). For one, this observation underlines how robust regions within a poorer context overconcentrate capital and activities at the expense of their surrounding regions; interestingly, the same applies even in Germany, France, and Denmark, although to a lesser extent. The above help us to (at least partially) refute theoretical and empirical postulations around the spillover effect of growth poles (see Florida *et al.*, 2008).

More importantly, the above highlight the importance of regional structures within a wider context of value chains, which brings us to the second factor of spatial unevenness in human capital distribution, **regional** **industrial specialisations**. The very constitution of “sequential structures” such as GVCs implies uneven development: some places get to host the “strong links” of these chains—i.e., the activities of higher value added which demand skilled labour—whereas the rest are left with the weaker ones ( Smichowski *et al.*, 2016). Within a value chain, a higher value added is typically attributed to the initial-stage and the later-stage activities, whereas those in the middle are lower value added (forming a “smiley face” curve; Mohiuddin & Su, 2014). Specifically, activities which come first in production chains relate to the standardisation, innovation, and design of a product, activities coming afterwards include the manufacturing and assembly of a product, and activities that come last relate to logistics, marketing, and branding ( Fróes de Borja Reis & Barroso de Souza, 2018). Thus, regions which for example host innovation research centres or constitute strong logistic centres are positioned in better terms within the global geography of skills, whereas those which constitute manufacturing/assembly centres are positioned in worse terms, except for the case of particularly high added value products ( Mohiuddin & Su, 2014). In sectoral terms, constructions, manufacturing, and the primary sector typically comprise lower skill level occupations and as a result attract workers with a lower education level ( Cedefop, 2018); in contrast, industries characterised by a high R&D expenditure/value added rate (e.g., biotech), as well as service and business operations stemming from scientific knowledge are linked with high-skill labour. In our analysis, regions standing out in terms of matching highly educated workers with high skill occupations included Île de France, Berlin in Germany, and Hovedstaden in Denmark. All of the above constitute innovative regions with a high GDP per capita – for its part, Île de France is an innovation leader at a pan-EU level (as indicated by the Regional Innovation Scoreboard; European Commission, 2022). Similarly, Yugozapaden, which also exhibited a notable high skill/education matching has taken significant steps towards economic modernisation and technological improvement in recent years ( European Commission, 2022).

As becomes evident, the first two factors discussed above are deeply interconnected. For one, regions cannot alter their industrial composition substantially without upgrading within global value chains. Moreover, this upgrading occurs usually within a wider context (i.e., the national one); it is not common for regions to upgrade while being part of obsolete domestic economic systems ( Gereffi & Lee, 2016). In the frame of our analysis, the overlapping of the first two factors can be indicated by the concentrations of highly skilled workers in middle skill level occupations, since this reflects some sort of skill mismatching. Specifically, all Greek regions displayed relevant overconcentrations, with Attica standing out among all study regions. This reflects not only Greece's weaker embeddedness within global value chains and lack of orientation towards productive activities (e.g., the North Aegean specialises in small-scale agriculture while the other insular regions overfocus on tourism; Herod *et al.*, 2022), but also its capital region being only but a moderate innovator, lagging significantly in terms of R&D spending ( European Commission, 2022). Of course, these industrial specialisations at the national and regional level cannot be seen outside a wider historical context, as contemporary global value chains are the product of long structural transformations and abrupt adaptations to crises. Specifically, in the aftermath of the crisis of Fordist industrialisation during the 1970s, regions in Northern and Central Europe took a turn toward technologically advanced sectors; in contrast, being unable to maintain their export capacity, peripheral regions across Southern Europe saw their industrial systems dismantled and resorted to less innovative, constructions-driven growth models ( Herod *et al.*, 2021). Additionally, the 2008/09 Global Crisis put a heavy strain on the peripheral parts of the EU and led to the burst of the real estate bubbles they had developed, decimating their construction activity ( Gourzis & Gialis, 2019). However, having their industrial structures already dismantled, these regions followed a course further into deindustrialisation, gradually developing a heavy dependence on tourism activities ( Herod *et al.*, 2022). As a result, their economic base in recent years comprises a mix of less innovative, technologically backward sectors such as construction, retail, and low value-added market services ( Fana *et al.*, 2016).

In further examining the determinants of skill (mis)matches, closely related to industrial specialisations is the **extent of urbanisation**. Regions with dynamic metropolitan areas typically abound in occupations requiring higher skill levels, thus, demand for skilled labour is high there ( Psycharis *et al.*, 2014); on the other end, urban environments often constitute more attractive environments for a high skilled and creative labour force as they are characterised by more amenities—cultural, transportations-wise, choices for education, etc.—and lower entry barriers for newcomers ( Florida *et al.*, 2008). As a result, our analysis indicated the highest levels of high education/skill matching in metropolitan regions. This is rather expected, since the main urban agglomerations within each national context act as dominant poles, accumulating population and capital, concentrating the better part of domestic infrastructure, and attracting foreign investments ( Perroux, 1955). In this frame, they develop economies of scale which fuel processes of mostly self-sustained growth ( Polyzos, 2015). Interestingly, our analysis found that instead of metropolitan regions in general, it was mostly the capital regions within each country that exhibited the aforementioned characteristics. On a deeper level, being the administrative and business centres in their national context, these regions represent their countries’ development potential; extending this postulation, their performance often determines a whole country’s positioning within global value chains ( Christofakis & Papadaskalopoulos, 2011). Nevertheless, we must note here that the above take place within a context of highly dualised labour markets; namely, besides the core of well-qualified people working in high skill occupations, there is a periphery of precarious workers undertaking menial tasks ( Teixeira, 2022). Lastly, we must also note that metropolitan regions’ potent forces of attraction lead to a skill mismatching-related paradox: our analysis indicated that super-star cities within robust national economies, as well as capital cities smaller national economies, concentrate a larger highly educated workforce than what their labour markets can sustain. As a result, they were found to display overconcentrations of highly educated people even in low-skill occupations, as in the case of Île de France. This is a particularly important observation, as it implies deep cutting inequalities and fierce competition even within the most dynamic regional labour markets, which exacerbate during recessive periods ( Herod *et al.*, 2021).

Nevertheless, such types of mismatches were found to be more prominent in less dynamic areas; it is characteristic that most of the regions at the periphery of the five study countries we studied here displayed concentrations of highly educated workers in low skill occupations. More importantly, in these regions, skill mismatches concerning the high-skilled labour do not solely emerge in times of economic turbulence, but they become their inherent, diachronic traits ( Iammarino *et al.*, 2019). A result of these widespread mismatching phenomena across various regional contexts is that one out of four university graduates in the EU work in jobs requiring lesser skills than what they have acquired through their education. This means that a good part of the workforce is underused ( Cedefop, 2018), despite approaches such as HCT postulating that overskilling cannot persist in the long run as it is automatically balanced by market mechanisms ( McGuinness & Pouliakas, 2016). Apart from a less advantageous position within domestic and international networks, a good part of these persistent skill mismatches should be attributed to weak **local institutional structures and practices**, which are apparent especially in peripheral regions ( Herod *et al.*, 2022). Specifically, weak institutions fuel ‘information asymmetries’ among tertiary education graduates leading them to poor career choices ( McGuinness *et al.*, 2018). The above observations are of crucial importance, as overskilling constitutes a serious threat, especially when it persists, since it undermines the incentive for young people to continue investing in their human capital ( Cedefop, 2015), diminishes productivity rates, and renders whole economy vulnerable to sudden changes ( Brunello & Wruuck, 2021). In addition to their weak institutional structures, peripheral regions also appear unable to attract and keep a minimum amount of high skilled labour to cover their needs ( Florida *et al.*, 2008), thus suffer from extensive underskilling as well ( Lee & Rodriguez-Pose, 2013). In this context, these regions enter a vicious cycle of being unable to fix skill mismatches, which in turn shapes the conditions that render those regions even more susceptive to all types of mismatches. This self-regenerating vulnerability comes in stark antithesis with the virtuous cycle of self-sustained growth that dynamic regions enter; combined, these cycles mark clear that—in the absence of decisive institutional interventions—spatial inequalities tend to deepen instead of being ameliorated over time ( Teixeira, 2022). 

One last decisive difference between urbanised and rural areas that has to be taken under consideration is their **demographic structure**. In Europe and elsewhere, urban centres retain a younger population as compared to rural areas ( Gregory & Patuelli, 2015). This shapes the composition of skills directly: older workers have lost their incentive to retrain, while the introduction of new technologies render their skill sets obsolete ( McGuinness *et al.*, 2018). At the same time, the weight of meeting skill demand falls upon the shoulders of those entering the labour market, who must develop the appropriate competencies ( Cedefop, 2015). Moreover, conditions of intensified competition further push younger people to invest more in their training ( Brunello & Wruuck, 2021), with education being their main defence mechanism in retaining their place in the job market (as postulated in the Job Competition Model; Sala, 2011). Such conditions are particularly prevalent in European labour markets post-2008 ( Gourzis & Gialis, 2019).

## Conclusions

This study examined the issue of human capital allocation across five European countries at the regional level, focusing on the regions where the university departments of the ERUA consortium are located: North and South Aegean in Greece, Île de France in France, Yugozapaden in Bulgaria, Freiburg in Germany, and Sjælland in Denmark. Our findings highlighted the uneven geography of skill (mis)matches, as capital and metropolitan regions were observed to be more efficient than less affluent and peripheral ones in matching supply (in terms of education) and demand (in terms of skill level occupations), especially when it comes to upper-level skills. To further decipher this remark, we discussed the role of five inter-related factors: economic dynamism at the national level, regional sectoral specialisations, urbanisation, institutional structures, and demographic trends. Importantly, our analysis pinpointed that human capital is shaped by multiple forces at the same time, most of which relate with each other in a dialectical way. Furthermore, we identified a series of virtuous and vicious cycles which drive uneven development as they refer to self-sustained, regenerating processes of resilience and vulnerability. Ultimately, we found human capital to be of the utmost importance in regional development; although it can generate spatial growth, its concentration in certain areas leaves lagging regions out of growth pathways.

To break this pattern, strong interventions are required. Thus, here we recommend a series of actions drawing upon the EU's New Skills Agenda, which are tailored for the regions associated with the ERUA but can be applied to other contexts as well. For one, in the regions with strong research and academic activity (as the ones related to the consortium), it is important that their universities work together with stakeholders from industry to spill over knowledge and cover the research and innovation needs of the labour market. In this frame, learning events can be developed to identify the skills needed in the labour market and communicate them to the unemployed and precariously employed. Moreover, these events must be combined with high quality career counselling for the unemployed and teaching of digital skills to the precariously employed, with special attention to vulnerable groups such as the youth, women, and migrants; this reskilling and upskilling could be undertaken by university staff in the frame of the European Competence Framework for Researchers. Apart from the above, social partners should be more encouraged to be involved in labour market forecasting and early identification of skill shortages and trends through joint actions; this way, a tighter network of social, scientific, and policy-related stakeholders would be formed. Finally, amid the current energy transition, developing green skills will produce a generation of climate-conscious professionals with pertinent competences that will ultimately benefit both the environment and the workforce.

## Ethical approval and consent

Ethical approval and consent were not required.

## Data Availability

Data used in this study are from the Eurostat’s Labour Force Survey dataset for all the European Union’s countries and for the years between 2008–2021. For the classification of occupations across levels, Eurostat follows the International Standard Classification of Occupations (ISCO-08) of the International Labour Organisation (ILO). For the classification of education levels, Eurostat follows the International Standard Classification of Education maintained by the United Nations Educational, Scientific and Cultural Organization. Access to the dataset requires registration and is granted only for legitimate research purposes. A guide for how to apply for dataset access is available at: https://ec.europa.eu/eurostat/documents/203647/771732/How_to_apply_for_microdata_access.pdf.

## Footnotes

^1^ International Labour Organisation-International Standard Classification of Occupations (ISCO) https://ilostat.ilo.org/resources/concepts-and-definitions/classification-occupation/
