# Supplementary material for: Investing in human capital in the era of European Universities: A comparative analysis of skill mismatches across the regions of the ERUA countries
Source: Open Res Eur. 2023 Oct 2;3:166. [Version 1] doi: 10.12688/openreseurope.16470.1 (PMC12658975; doi:10.12688/openreseurope.16470.1)
Supplement: Peer review history

# Reviewer report: Reviewer response for version 1

**DOI:** 10.21956/openreseurope.17779.r38002

**Published:** 2024-04-19

**Authors:** Alexander Patt

**Affiliations:** 1 Institute for Employment Research, Nürnberg, Germany

The paper offers a perspective on regional allocation of human capital and skill mismatch by discussing different strands of the literature (human capital theory, economic geography, education policy, institutions, etc.) and showing complementary empirical evidence using variation in the location quotient index. The index is based on education shares across three broad occupational groups in NUTS2 regions from a selected group of countries. The authors document considerable regional variation in skill mismatch.

 Overall, I found the paper thought-provoking and I appreciated that it drew upon various literatures. However, I am unsure that it is possible to adequately address all questions raised by the paper within the analysis framework chosen by the authors. More specific comments follow.

 i) The paper does not separate normative from positive statements in a clear way, while lacking notion of an optimal allocation of talent without which such discussion cannot be done. In the empirical part, the authors focus on vertical mismatch, by which they implicitly mean high skilled workers employed in non-managerial and non-professional roles (ISCO groups below 3). However, such allocation could arise due to a particular industry structure which favours middle-skilled occupations and therefore attracts a disproportionate amount of workers across all skill levels. The paper could be improved by separating the issues of spatial allocation from mismatch, and by corroborating the results on mismatch by other indicators of skill shortages and oversupply, such as rates of return to education, local unemployment rates and job turnover.

 ii) The authors' construction of the quotient index assumes that all countries should have similar relative shares of human capital allocation, but this ignores country-level differences in education systems organisation and differences in economic development (as more developed economies tend to have higher investment rates in education in general). For example, vocational education plays a relatively larger role in Germany and provides educational tracks to occupations which would otherwise require a college degree in other countries. Since such country-level differences make the results difficult to interpret, the authors should focus on within-country variation.

 iii) While the paper introduces five different factors of regional specialisation, the empirical approach doesn't in fact leave the realm of the neoclassical human capital theory, as it is largely informed by spatial allocation of workers within the standard labour demand framework with regional specialisation. While the authors mention other determinants such as global value chains and local institutions, there is no systematic characterisation of their variation shown with data. The findings on mismatch in particular lack an interpretation supported by documented evidence on country and regional differences along the dimensions considered in the paper. Given limitations of regional data, it is doubtful that it is feasible to discuss all these mechanisms in detail and isolate their effects. However, the paper can still be informative by developing comprehensive descriptive evidence in a systematic way.

 iv) The paper omits critical details about the sample construction: it is not clear whether the authors imposed age restrictions, used only full-time or part-time workers, included both genders or only males, included or excluded migrant workers (which can have strong implications for the results). It's also not clear whether nation averages of the shares are weighted. How did the authors handle missing data?

 v) I am concerned about specific years chosen for the analysis (2009, 2021) which could affect the results due to strong negative economic shocks which were unevenly distributed. Since the analysis doesn't rely on time variation, the authors should use different years and average the results across 5 or 10 year intervals to reduce noise.

 vi) The paper only shows the results for high skilled workers. What about other groups?

If applicable, is the statistical analysis and its interpretation appropriate?

No

Is the study design appropriate and is the work technically sound?

No

Is the work clearly and accurately presented and does it engage with the current literature?

Partly

Are the conclusions drawn adequately supported by the results?

No

Are sufficient details of methods and analysis provided to allow replication by others?

Partly

Are all the source data and materials underlying the results available?

Yes

Reviewer Expertise:

Labour economics

I confirm that I have read this submission and believe that I have an appropriate level of expertise to state that I do not consider it to be of an acceptable scientific standard, for reasons outlined above.

# Response

**Published:** 2025-04-08

**Authors:** Effie Emmanouil

**Affiliations:** Geography, University of the Aegean, Mytilene, Greece, Greece

Dear Reviewer, we would like to thank you for your valuable and insightful comments. We have proceeded with major revisions on our manuscript, following most of your points, which we found well raised. These changes have bolstered our arguments, slightly extending the text’s wording, without changing its meaning. Please find below our responses to your comments in detail.  

**Reviewer: The paper does not separate normative from positive statements in a clear way, while lacking the notion of an optimal allocation of talent without which such discussion cannot be done.**

**Author Response:** To address the first part of this crucial comment, we rephrased various sections of the manuscript. Now, observations based on empirical evidence are clearly stated, allowing the reader to easily distinguish them from statements which express our opinions. Regarding the lack of the notion of an optimal allocation of talent, we have revised the Theoretical and Conceptual Section of our manuscript. Specifically, in this section we open a whole part where we discuss the factors shaping the uneven geography of skill mismatches writing that: *“To maximize the effects of higher educational attainment, however, talent has to be allocated in an optimal way, namely, a worker must employ their skills in the most appropriate setting. It is then through the optimal allocation of talent that a higher educational attainment level generates individual and social returns (Acemoglu, 1995).”* Further below, the concept is scrutinized in detail, using the appropriate literature. Then, in the Discussion, the concept now plays a key role.  

**In the empirical part, the authors focus on vertical mismatch, by which they implicitly mean high skilled workers employed in non-managerial and non-professional roles (ISCO groups below 3). However, such allocation could arise due to a particular industry structure which favours middle-skilled occupations and therefore attracts a disproportionate amount of workers across all skill levels. The paper could be improved by separating the issues of spatial allocation from mismatch, and by corroborating the results on mismatch by other indicators of skill shortages and oversupply, such as rates of return to education, local unemployment rates and job turnover.**

**Author Response: **This is a well raised point. However, due to limited space, we could not address both the issues of mismatches’ spatial allocation as well as the nature of vertical mismatches. Unavoidably, we relied on secondary data that do not consider the qualitative aspects of these vertical mismatches. Nevertheless, we linked the concentration of various types of skill (mis)matches with a series of factors, including through macroeconomic, population, innovation, and demographic indicators. Please refer to our response to Comment 8 by Reviewer 1. The issue of  particular industry structures which favour middle-skilled occupations is addressed in the Discussion. Indicatively, we write: *“Apart from tourism, there are many manufacturing- and assembly-related activities along GVCs that favor medium skill-level occupations and therefore generate tendencies towards a disproportionate amount of highly educated workers in such occupations. Such is the case with most French regions, and especially the agri-food heavy Franche-Comté.”*  

**While the paper introduces five different factors of regional specialisation, the empirical approach doesn't in fact leave the realm of the neoclassical human capital theory, as it is largely informed by spatial allocation of workers within the standard labour demand framework with regional specialisation. While the authors mention other determinants such as global value chains and local institutions, there is no systematic characterisation of their variation shown with data. The findings on mismatch in particular lack an interpretation supported by documented evidence on country and regional differences along the dimensions considered in the paper. Given limitations of regional data, it is doubtful that it is feasible to discuss all these mechanisms in detail and isolate their effects. However, the paper can still be informative by developing comprehensive descriptive evidence in a systematic way.**

**Author Response: **It is correctly stated that limitations of regional data, as well as the paper’s word limitation, did not allow us to make our postulations regarding GVCs more evidence-based. Nevertheless, we addressed your comment by developing comprehensive descriptive evidence in a systematic way around the five factors inquired in the Discussion. Specifically, our Empirical Analysis now provides concrete evidence for our postulations in the Discussion by including a scrutiny of indicators on GDP, innovation performance, population density, and demographic structures. Please refer to our response to Comment 8 by Reviewer 1 for more detail regarding the proxies that we used and the methods we implemented for their analysis. Moreover, all these factors are covered in the Theoretical and Conceptual Section as well. Please refer to our response to Comment 3 by Reviewer 2 for more detail.  

**The authors' construction of the quotient index assumes that all countries should have similar relative shares of human capital allocation, but this ignores country-level differences in education systems organisation and differences in economic development (as more developed economies tend to have higher investment rates in education in general). For example, vocational education plays a relatively larger role in Germany and provides educational tracks to occupations which would otherwise require a college degree in other countries. Since such country-level differences make the results difficult to interpret, the authors should focus on within-country variation.** **Author Response: **Although this is a well raised point, the main goal of our analysis was to directly compare the performance of various EU regions in terms of addressing skill mismatches. For that reason, we went with the relative-to-transnational LQ as our main index. Nevertheless, we accept that structural differences between countries make such comparisons a difficult case. Apart from the latter, we are perfectly aware that the LQ can sometimes lead to contradicting results due to not providing a clear view of the absolute magnitude of the phenomenon under study, or due to the modifiable areal unit problem. To overcome all the above, we tried to consider a wide array of country-specific factors when discussing our findings. For instance, we have extensively relied on relevant research around the employment/educational regimes typology. For instance, we write in the revised Theoretical and Conceptual Section: *“Nevertheless, dual educational systems such as Germany’s have also been praised for their positive effects in school-to-work transitions, albeit having been oversimplified in the relevant literature (Deissinger, 2015). Similarly praised is the universalistic regime in Nordic countries like Denmark, which invest in human capital and match high levels of educational attainment with demand for such workers (Hadjivassiliou et al., 2019). [...] …in statist regimes, for instance, employers are not sufficiently involved in matching skills’ demand and supply (Capsada-Munsech & Valiente, 2020). As such, in the sub-protective regime of the Mediterranean EU, in countries like Greece, youth transitions to adulthood are long and problematic (Emmanouil et al., 2023). This leads to a large pool of highly qualified people who either cannot find a job, have a profession for which they are overqualified, or have a profession in a different sector from the one in which they were trained, while at the same time many companies are struggling to fill their vacancies (Biagi et al., 2020).”* Similarly, in the Discussion we write: *“...the universalistic (e.g.Denmark) regime with high investments in education and fast and stable school-to-work transitions is completely different from countries of the sub-protective regime (e.g. Greece), which display severe skills mismatches and low labour demand in general (Hadjivassiliou et al., 2019).”.*

**The paper omits critical details about the sample construction: it is not clear whether the authors imposed age restrictions, used only full-time or part-time workers, included both genders or only males, included or excluded migrant workers (which can have strong implications for the results). It's also not clear whether nation averages of the shares are weighted. How did the authors handle missing data?**

**Author Response: **To address this comment, we have added to the Methodological Section that *“Eurostat’s sample covers all employed persons of both genders, aged 15 and above. Data imputation methods were not used, given that the extent of missing data is limited and most missing values refer to a specific type of skill mismatch (highly educated workers in low skill-level occupations), making most such methods unreliable.” *  

**I am concerned about specific years chosen for the analysis (2009, 2021) which could affect the results due to strong negative economic shocks which were unevenly distributed. Since the analysis doesn't rely on time variation, the authors should use different years and average the results across 5 or 10 year intervals to reduce noise.** **Author Response: **This is another excellent point which could lead to very interesting findings. In practical terms, we could add one or two years between 2009 and 2021. However, this could substantially add to the wordage of the manuscript. Moreover, we believe that the years under study as it is allow for a comparison between two crucial moments in the EU’s recent history: the onset of a period of successive crises and the onset of a period of a renewed round of pandemic- and geopolitically-induced crises. Moreover, please note that our analysis, given that the LQ is a relative indicator, avoids looking into the changes in the extent of skill mismatches between 2009 and 2021. Relatedly, we note in the Empirical Analysis Section: *“It must be noted that the LQ is a relative index, meaning that its changes over time do not indicate neither absolute growth nor retraction of the phenomenon under study. Therefore, correlations pertaining to LQ changes over time must be approached with caution.”* As such, we believe that the noise does not affect the significance of our observations considerably.

# Reviewer report: Reviewer response for version 1

**DOI:** 10.21956/openreseurope.17779.r36271

**Published:** 2024-01-02

**Authors:** Martin Henning

**Affiliations:** 1 Department of Business Administration, University of Gothenburg, Göteborg, Sweden

This article approaches a very interesting and important topic: the geography of skill mismatches in Europe. The article uses quantitative data to map skill mismatches in selected regions, and discusses reasons as to why these geographical variations arise.

 In fact, it would benefit the article if it could focus *even more* on this phenomenon and its explanations. The introduction is interesting because it juxtaposes more traditional human capital theory with arguments derived from geographical political economy. However, while one could debate the outcomes of this intellectual tension, this is not really the core of the article (human capital theory is though dismissed as simplistic and positivist – a language I would myself avoid given the convincing literature on the overall benefits of education and higher skills). The focus of the article is not to estimate the benefits from education. Instead, I would wish for a stronger focus on the *geography of skill mismatches* in the introduction. This also would open to the real empirical geographical question in the article – what does the geography of skill mismatches look like, and why do differences between regions arise across Europe?

 I would also recommend a revision of the literature review in the same fashion (section: Human capital and its uneven spatial distribution). Right now, this section focuses on human capital theory and some sources of mismatches. The discussion makes a valid point around the importance of institutions (in fact, one could probably argue that labour market and education institutions are among the most variable factors across the countries within the single market). Moreover, the geographical discussion treats the issue of regional divergence and uneven regional development. On this topic, there is already a sizeable literature, and this should also not be the core discussion of this paper. While I would actually like to challenge some of the arguments in a within-European context (for example, that growth is a trade-off between regions, the peculiar connection made between vocational training and precarious labour and the new rounds of “Fordist-influenced spatial organization” – within Europe the Fordist era was in fact characterized by decreasing regional economic disparities), this is besides the point.

 Instead, I would like to recommend a stronger focus on the geographies of mismatches and why they arise. The paper would make a stronger contribution if it could remain with this focus. In particular, I would welcome a discussion about the dimensions that the authors later return to as explaining the geography of mismatches: dynamism of national economies, regional specializations, local institutions and demographic structure. In such a context, for example, the discussion about the global production chains and their spatialities is interesting, but it does not quite explain the geography of mismatches.

 In the empirical part, the use of the different LQs is ingenious. However, it would be great if the authors could comment on the reliability of the mismatch indicator: is there a risk that we simply misjudged the level of skills needed in jobs, or that they changed? And where in the economy do the most important mismatches arise? I believe that this could be easily described using the available data, as an introduction to the geographical analysis. It would also be good if the authors explained whether the patterns could be taken to be general – or are the results a feature of the regions/countries that selected themselves into the university initiative?

  The authors caution a warning for fig. 1.1a and b. It is hard what to make of this – is this part of the analysis reliable or not?

 In the discussion section, the authors are looking for different ways to explain the geographical variation in the mismatches. It would be good to know how the authors ended up thinking about these factors (linking to the theoretical section above). Secondly, it is a bit hard for the reader to evaluate the relevance of the factors. Here, I would recommend the authors to provide empirical evidence. If not in terms of regressions, so at least displaying the co-variation between the factors and mismatches in regions. For example, one could display a scatterplot between the size of the mismatch in regions, and the demographic structure. I believe that relevant indicators could be calculated for all the dimensions. Such an exercise would make the arguments much more convincing and interesting.

 To complement this, we should always keep in mind that the LQs illustrate the spatial variation of a phenomenon (relative), and not how important the phenomenon itself is (absolute). In fact, it would be good to know the absolute level too – how sizeable are the mismatches around European regions? This would be a way to illustrate the absolute importance of the phenomenon. Also, but this is maybe too much of an extension, it would be very interesting to know if the mismatches actually have consequences for regional growth (or, are the mismatches rather a result of growth because growing regions attract labour that maybe cannot be accommodated immediately?). Using the lag structure of the data, such an experiment could be made.

 In the policy recommendations, the authors argue for “strong interventions”. However, the interventions that they mention seem to be only vaguely related to the findings of this paper (except maybe for the high-quality career counselling). Instead, I would encourage the authors to develop policy recommendations that are immediately associated with the findings of this paper.

 In sum, I find the topic and empirical approach very promising. However, revisions according to the above would give the paper a much stronger basis to argue for particular and precise conclusions concerning the geography of skill mismatches in the European Union. I do hope that the authors find these comments constructive for further work.

If applicable, is the statistical analysis and its interpretation appropriate?

Partly

Is the study design appropriate and is the work technically sound?

Partly

Is the work clearly and accurately presented and does it engage with the current literature?

Partly

Are the conclusions drawn adequately supported by the results?

No

Are sufficient details of methods and analysis provided to allow replication by others?

Not applicable

Are all the source data and materials underlying the results available?

Yes

Reviewer Expertise:

Economic geography

I confirm that I have read this submission and believe that I have an appropriate level of expertise to state that I do not consider it to be of an acceptable scientific standard, for reasons outlined above.

# Response

**Published:** 2025-04-08

**Authors:** Effie Emmanouil

**Affiliations:** Geography, University of the Aegean, Mytilene, Greece, Greece

Dear Reviewer, we would like to thank you for your valuable and insightful comments. We have proceeded with major revisions on our manuscript, following most of your points, which we found well raised. These changes have bolstered our arguments, slightly extending the text’s wording, without changing its meaning. Please find below our responses to your comments in detail.

1. **The article uses quantitative data to map skill mismatches in selected regions, and discusses reasons as to why these geographical variations arise.** **In fact, it would benefit the article if it could focus even more on this phenomenon and its explanations. The introduction is interesting because it juxtaposes more traditional human capital theory with arguments derived from geographical political economy. However, while one could debate the outcomes of this intellectual tension, this is not really the core of the article (human capital theory is though dismissed as simplistic and positivist – a language I would myself avoid given the convincing literature on the overall benefits of education and higher skills). The focus of the article is not to estimate the benefits from education. Instead, I would wish for a stronger focus on the geography of skill mismatches in the introduction. This also would open to the real empirical geographical question in the article – what does the geography of skill mismatches look like, and why do differences between regions arise across Europe?**

This is a well raised point. In the revised version of the manuscript, we clarify our empirically-grounded positioning in relation to the HCT. Overall, we try to show that a fruitful synthesis of notions from HCT with notions from GPE can be a very illuminating theoretical and practical exercise that sheds light on the deeper factors driving skill mismatches. Apart from the above, we follow your suggestion and enhance our focus on the uneven geography of skill mismatches. In doing so, we have advanced the aim and the specific questions in the Introduction but also have adapted the subsequent sections accordingly. Especially in the Theoretical and Conceptual Section, we now focus more on the socio-spatial drivers of skill mismatches and explain the reasons why geographical variations arise. This is detailed when addressing your second point. Lastly, in the Introduction we put more emphasis on what the EU is doing to address skill mismatches.  

1. **On this topic, there is already a sizeable literature, and this should also not be the core discussion of this paper. While I would actually like to challenge some of the arguments in a within-European context (for example, that growth is a trade-off between regions, the peculiar connection made between vocational training and precarious labour and the new rounds of “Fordist-influenced spatial organization” – within Europe the Fordist era was in fact characterized by decreasing regional economic disparities), this is besides the point. Instead, I would like to recommend a stronger focus on the geographies of mismatches and why they arise. The paper would make a stronger contribution if it could remain with this focus. In particular, I would welcome a discussion about the dimensions that the authors later return to as explaining the geography of mismatches: dynamism of national economies, regional specializations, local institutions and demographic structure. In such a context, for example, the discussion about the global production chains and their spatialities is interesting, but it does not quite explain the geography of mismatches.**

This is another well raised point that we have taken into account in our revisions. Specifically, the Theoretical and Conceptual Section has been extensively revised so as to follow a clear narrative revolving around the uneven geography of skill mismatches. In this section then, we now present some basic points raised in the literature regarding the factors underlying this uneven geography; to do so, we have added numerous new sources. As you stressed, most of these points pertain to the factors that are inquired in the Discussion Section (dynamism of national economies, regional productive priorities, institutional endowments, urbanization levels, etc.). To deepen this Section’s clear targeting, we have also taken out a good part of the postulations regarding the effect of Fordism on uneven development, as well as the role of global value chains. The same principle is followed in the Discussion.   

1. **In the empirical part, the use of the different LQs is ingenious. However, it would be great if the authors could comment on the reliability of the mismatch indicator: is there a risk that we simply misjudged the level of skills needed in jobs, or that they changed?**

To clarify the source for our calculated LQs, we now stress in the Methodological Section: *“Data on the matching between highly educated workers and low, medium, and high skill-level occupations are directly calculated by Eurostat,  based on its Labour Force Survey.”* Further below, we now add in regard to the reliability of the mismatch indicator: *“The classification of skill levels was carried out at our request by Eurostat itself, to eliminate the risk of misjudging skill levels and to increase the reliability of the mismatch index.”* It should be clear then, that the shares of highly educated workers per skill-level occupation at the regional level were directly provided to us by Eurostat. Of course, Eurostat’s methodology carries some inherent weaknesses, such as that skill mismatches are judged solely based on the correspondence between a worker’s education attainment level (following the ISCED typology) and an occupation’s demanded skill level (following the ISCO typology). No qualitative dimensions are then taken into account; but this is a shortcoming characterizing most—if not all—secondary analyses on skill mismatches (as noted by several experts in the literature; e.g., see MacGuinness *et al.*, 2018).  

1. **And where in the economy do the most important mismatches arise? I believe that this could be easily described using the available data, as an introduction to the geographical analysis. **

We must note that this is another well raised point that would lead to very interesting findings. However, we have not inquired about skill mismatches by sector, for two main reasons. First, for reasons of data limitations. After checking the available data, analysis per sector and region would have to rely on really small samples which would not be reliable. This is stated in the Methodology Section: *“...as the focus of this paper is on the regional level, our analysis cannot include skill mismatches by sector and region, given that this would lead to extensive data fragmentation and therefore to issues of reliability and missing values.”* Secondly, a sectoral analysis would require the addition of substantial text, which would make the manuscript even lengthier. Therefore, we deemed that a sectoral analysis—albeit interesting—lies outside the scope of our paper. Nevertheless, we have included relevant insight from the literature in the Theoretical and Conceptual Section, and then revisit this insight in the Discussion. Specifically, in the former section we write: *“...apart from the dynamism of a regional industrial structure, the sectoral specialization per se plays a key role. Characteristically, sectors like accommodation and catering, commerce and storage, and transportation,  are reported to suffer greater skill mismatches—both horizontal and vertical—than others (Chłoń-Domińczak & Żurawski, 2017).”*

1. **It would also be good if the authors explained whether the patterns could be taken to be general – or are the results a feature of the regions/countries that selected themselves into the university initiative?**

Although our study focuses mainly on the ERUA regions and countries, our findings can be considered more broadly. We state the reason for this in the Methodological Section, which is that the countries under study were chosen not only because of the ReERUA Project, but also because they represent all key educational/employment regimes in Europe. Specifically, we write: *“The analysis is conducted at the regional level (NUTS-2) and its geographical scope covers five countries: Greece, Bulgaria, France, Germany, and Denmark. These were chosen on the basis that they represent the main educational/employment regimes in Europe: the universalistic of the Nordic countries, the employment-centered of Continental Europe, the sub-protective of the Mediterranean countries, and the post-socialist of Eastern Europe (Pohl & Walther 2007; Farkas, 2011). While both France and Germany fall under the Continental model, they are both analyzed due to their stark differences in terms of their wage structures (Hein & Vogel, 2009) and educational models (O’Reilly et al., 2019). Given the research’s geographical scope covering all main European regimes pertaining to employment and skills, its findings can be contextualized more broadly and provide insight into the EU as a whole.”* The above is also implied in the Introduction and the Discussion.  

1. **The authors caution a warning for fig. 1.1a and b. It is hard what to make of this – is this part of the analysis reliable or not?**

This is an extremely important point. To address it and secure the reliability of our analysis, we now clarify in the Empirical Analysis Section that: *“Paradoxically, in both Figures 2a and 2b, LQ scores fluctuate exclusively above 1, indicating over-concentrations in all regions for which data were available. The reason for this is because the phenomenon of highly educated workers being employed in low skill-level occupations is inherently limited. As a result, regions with a small number of such workers, which would exhibit LQ scores below 1, have a non-statistically significant sample, thus data for them is missing. Conversely, the available data allows for observing where highly educated workers are over-concentrated in low skill-level occupations.”* In other words, our observations regarding where highly educated workers in low skill-level occupations are concentrated, we follow a process of elimination. Namely, although we do not have sufficient data to directly observe which regions exhibit under-concentration of this type of skill mismatch, we systematically exclude all other possibilities based on the available data, and we are left with the answer to our problem.  

1. **In the discussion section, the authors are looking for different ways to explain the geographical variation in the mismatches. It would be good to know how the authors ended up thinking about these factors.**

  To address this comment, we have revised several sections of our manuscript. In short, we can state here that we ended up with these based on the insight of the relevant literature. This was the case from the start; however, it is a fact that this was not adequately clarified in our initial submission. Thus, including them in the Theoretical and Conceptual Section was crucial. Doing so, we cover the literature around the role of the dynamism of national economies in skills’ geography in a paragraph starting with: *“Various factors shape the widely documented uneven geography of skills and their mismatches (Alamá-Sabater et al., 2024; Cedefop, 2010). Importantly, regional mismatches can be greatly affected by the robustness (or lack thereof) of the broader national economy.”* Similarly, we write about the effect of regional specializations: *“At the regional level, the dynamism of an economy is equally crucial. Capital- and technology-intensive activities tend to concentrate in already advanced regions, fueling new rounds of uneven development (Vinuela-Jimenez et al., 2010). As such, metropolitan areas with a diverse economic base concentrate human capital, cutting-edge technology, and finance-related activities, while economically backward and declining regions become even more disconnected from knowledge and innovation (Feldman et al., 2021). Similarly, developed industrial regions tend to attract new activities, workforce, and capital, as they further industrialise (Boudeville, 1968), although in many cases they also spill over positive effects to their surrounding areas (Perroux, 1955).”* The role of institutional endowments is scrutinized throughout this section, as for instance in the part we write: *“Such segmentation is crucially sustained by dual human capital development mechanisms. Namely, next to a robust education system, a training system that focuses more on vocational training and apprenticeship initiatives is formed, serving as a channel for precarious labour (Teixeira, 2019).”* Apart from the above, we lay out the relevant literature’s insight into the effect of crises of various types on the geography of skill mismatches in the paragraph starting with: *“Beyond the national and regional factors, sudden shocks of economic or other nature, as well as structural transitions, directly contribute to increasing skill mismatches (Cedefop, 2015).”* The latter is particularly important in justifying our rationale behind the temporal scope of our paper covering a period of successive crises. Then, in the Empirical Analysis these crucial factors are scrutinized  through a series of correlations between our LQs and proxies. Relatedly, we state in the Methodological Section: *“Regarding the correlation analysis, the research at hand statistically associates the LQ values of the above analysis with crucial factors underlying the geographical allocation of skills as noted in the relevant literature (e.g. Cedefop, 2010). Specifically, it links the LQ* *L* *, LQ* *M* *, and LQ* *H* *with regional and national GDP (both total and per capita), the European Commission’s Regional Innovation Index, urbanization levels, the Ageing Index, and the Dependency Index (see Table 1).”* Further below, in the beginning of Discussion, we stress this again: *“Drawing upon our main findings, here we discuss the most crucial factors underlying the uneven geography of skill mismatches. In this framework, we put particular emphasis on the economic dynamism of national economies, regional industrial structures, urbanization, local institutional structures and practices, and demographic structures, drawing from the main findings of the relevant literature (Alamá-Sabater, 2024; Cedefop, 2010).”  *  

1. **Secondly, it is a bit hard for the reader to evaluate the relevance of the factors. Here, I would recommend the authors to provide empirical evidence. If not in terms of regressions, so at least displaying the co-variation between the factors and mismatches in regions. For example, one could display a scatterplot between the size of the mismatch in regions, and the demographic structure. I believe that relevant indicators could be calculated for all the dimensions. Such an exercise would make the arguments much more convincing and interesting.**

To address this crucial comment, which was among the most important we received, we proceeded with major revisions on our manuscript. Specifically, following your suggestions, we added a correlation analysis (through Pearson’s R) to our implemented methodologies. Specifically, bivariate correlations have been conducted between the LQ _L_, the LQ _M_, and the LQ _H_ on the one hand, and the national and regional GDP (total and per capita), a regional Innovation Index by the European Commission, population density representing the level of a region’s urbanisation, as well as the Ageing Index and the (labour force) Dependency Index pertaining to a region’s demographic structures. The specifics of this analysis (temporal scope, methods, data sources, interpretation approach, etc.) are laid out in detail in the last paragraph of the Methodological Section. Then, in the Empirical Analysis section, the correlation analysis follows the existing LQ analysis so as to contextualize its findings. The results of the correlation analysis are presented in detail in a correlation matrix (Table 1). We believe that with the above we make our arguments in the Discussion more convincing, since they are now clearly evidence-based.  

1. **To complement this, we should always keep in mind that the LQs illustrate the spatial variation of a phenomenon (relative), and not how important the phenomenon itself is (absolute). In fact, it would be good to know the absolute level too – how sizeable are the mismatches around European regions? This would be a way to illustrate the absolute importance of the phenomenon.**

Please note that apart from the LQs that are presented in Figures 2-4, we also include a table in the Annex that displays all raw data based on which we calculated the LQs. Specifically, in this table we lay out the shares of highly educated workers per skill-level occupation within each region under study. This way, the reader has a clear view of the magnitude of the phenomenon under study. We also considered including the absolute volumes of the above, however, that would make the table much bigger, which we decided to avoid.   

1. **Also, but this is maybe too much of an extension, it would be very interesting to know if the mismatches actually have consequences for regional growth (or, are the mismatches rather a result of growth because growing regions attract labour that maybe cannot be accommodated immediately?).**

To address this comment, we have included in our correlation analysis the regional GDP. Based on the findings of this analysis, we draw a series of findings on the link between skill mismatches and regional growth. Specifically, regarding LQH, we note in the Empirical Analysis Section: *“The above findings indicate that perfect skill matching can mainly be observed in regions with robust economies, regardless if they are located in countries that are not as economically dynamic.”* Further below, we write about LQM: *“...it is found that productive robustness at the national level affected this type of skill mismatch more than robustness at the regional level did.”* Lastly, regarding LQL, we write: *“...there are many regions within otherwise robust national economies that exhibited extreme overskilling, as well as regions within meagre national economies with optimal skill matches.”*  

1. **In the policy recommendations, the authors argue for “strong interventions”. However, the interventions that they mention seem to be only vaguely related to the findings of this paper (except maybe for the high-quality career counselling). Instead, I would encourage the authors to develop policy recommendations that are immediately associated with the findings of this paper.**

In the revised version of the text, we toned down the statement about “strong interventions”, writing instead that: *“To break this pattern of uneven development, policy interventions are required.”* Nevertheless, in the revised Conclusions policy recommendations are more targeted and concrete, drawing directly from the findings of the Empirical Analysis. Specifically, they revolve around the significance of: knowledge spillover and alignment between education and innovation needs; entrepreneurial activity rechanneling into underdeveloped regions; and lagging regions’ re-specialization in more dynamic high value-added and high-skill sectors. For each one of those, we provide specific examples and/or cases.
